# Effects of an Additional Sports Gymnastics Program on Sagittal Spinal Alignment and Postural Status in School-Aged Students

**DOI:** 10.3390/jfmk11030270

**Published:** 2026-07-14

**Authors:** Raid Mekić, Stefan Đorđević, Oliver Radenković, Vladan Milić, Ilma Čaprić, Benin Murić, Milovan Bratić, Izet Kahrović, Omer Špirtović, Rifat Mujanović, Elvis Mahmutović, Mia Stanojević, Zoran Bajin

**Affiliations:** 1 Department of Biomedical Sciences, State University of Novi Pazar, 36300 Novi Pazar, Serbia; rmekic@np.ac.rs (R.M.); vmilic@np.ac.rs (V.M.); icapric@np.ac.rs (I.Č.); bmuric@np.ac.rs (B.M.); bratic_milovan@yahoo.com (M.B.); ikahrovic@np.ac.rs (I.K.); ospirtovic@np.ac.rs (O.Š.); rmujanovic@np.ac.rs (R.M.); ehmahmutovic@np.ac.rs (E.M.); zbajin@np.ac.rs (Z.B.); 2 Faculty of Sport and Physical Education, University of Niš, 18000 Niš, Serbia; 3 Faculty of Sport and Physical Education, University of Novi Sad, 21000 Novi Sad, Serbia; stanojevicmia6@gmail.com

**Keywords:** sports gymnastics, sagittal spinal alignment, spinal curvature, spinal mobility, kyphosis, lordosis, school children, physical education

## Abstract

**Background**: Sagittal spinal alignment is an important indicator of musculoskeletal health during childhood. Although sports gymnastics has been suggested as a potentially beneficial form of physical activity for postural development, evidence from short-term school-based interventions remains limited. Therefore, the aim of this study was to examine the effects of an additional sports gymnastics training program on sagittal spinal alignment in fifth-grade primary school students. **Methods**: A total of 139 fifth-grade students (11.0 ± 0.5 years) participated in this quasi-experimental longitudinal study and were allocated to an experimental group (*n* = 74) and a control group (*n* = 65). The intervention lasted six weeks and consisted of one additional sports gymnastics session per week (60 min) in addition to regular physical education classes. Sagittal spinal alignment was assessed using the Spinal Mouse device by measuring thoracic kyphosis and lumbar lordosis. Baseline-adjusted between-group differences were evaluated using analysis of covariance (ANCOVA). **Results**: Small descriptive reductions in thoracic kyphosis and lumbar lordosis were observed in the experimental group; however, these within-group changes did not reach statistical significance. After adjustment for baseline values, ANCOVA demonstrated a statistically significant between-group difference for thoracic kyphosis (F = 5.05, *p* = 0.026, partial η^2^ = 0.038). Nevertheless, because the adjusted mean value was lower in the control group than in the experimental group, this finding should be interpreted with caution. No significant between-group difference was observed for lumbar lordosis (F = 2.24, *p* = 0.137, partial η^2^ = 0.017). An increase in the proportion of participants classified as having physiological sagittal spinal alignment was observed in both groups, with a descriptively greater increase in the experimental group (14.9% to 45.9%) than in the control group (7.7% to 35.4%). **Conclusions**: The findings provide preliminary evidence that a short-term additional sports gymnastics program may be associated with changes in sagittal spinal alignment among school-aged children. However, because of the quasi-experimental design, the low intervention dose, and the short duration of the program, the results should be considered exploratory. Further randomized controlled studies with longer intervention periods are needed to clarify the effects of sports gymnastics on sagittal spinal alignment in children.

## 1. Introduction

Modern lifestyle habits, characterized by prolonged sitting time and reduced levels of daily physical activity, significantly contribute to impaired spinal alignment among school-aged children [1,2,3,4]. Prolonged maintenance of non-physiological postures may lead to muscular imbalance and inappropriate loading of the spinal column, most commonly manifested as alterations in thoracic kyphosis and lumbar lordosis in the sagittal plane [5,6]. If not recognized and addressed in a timely manner, these deviations may progress into structural deformities with long-term consequences for functional capacity and overall health [7,8]. Proper sagittal spinal alignment is characterized by physiological values of thoracic kyphosis and lumbar lordosis, ensuring optimal load distribution and efficient maintenance of body balance. Objective assessment of these parameters is particularly important during school age, as it enables early identification of deviations and implementation of appropriate corrective interventions [9,10,11].

Physical education represents an important component of the educational system, aimed at promoting healthy physical development, preserving health, and fostering lifelong physical activity habits among students [12]. During periods of intensive growth and development, the locomotor system exhibits substantial plasticity but also increased susceptibility to external influences, further emphasizing the importance of physical education in the prevention and correction of spinal deviations [13,14,15,16,17,18].

In this context, sports gymnastics represents a complex motor activity that incorporates exercises aimed at developing trunk strength, flexibility, coordination, and spinal stabilization. Through symmetrical activation of muscle groups and high demands for body control, gymnastics-based activities may contribute substantially to improvements in sagittal spinal alignment and reductions in spinal deviations [19,20,21,22].

Epidemiological evidence indicates a high prevalence of spinal deviations among school-aged children. Only 38.1% of students demonstrate physiological spinal alignment, whereas kyphotic and lordotic deviations are present in 7.6% and 1.0% of cases, respectively [23]. Furthermore, spinal irregularities in both the sagittal and frontal planes have been reported in approximately 32% of boys aged 7–15 years [24]. The problem appears even more pronounced among children with excessive body weight, in whom kyphotic posture has been observed in 41.1% and lordotic posture in 61.7% of cases [25], suggesting a possible relationship between body composition and sagittal spinal curvature.

Although previous studies have reported beneficial effects of organized physical activity and gymnastics-based programs on postural characteristics and sagittal spinal alignment [26,27,28], several important methodological limitations remain. First, only a limited number of studies have quantitatively assessed thoracic kyphosis and lumbar lordosis using valid instrumental methods such as the Spinal Mouse. Second, most intervention programs have involved considerably higher training volumes (two to five sessions per week), which are difficult to implement within regular school-based physical education. Consequently, evidence regarding the effectiveness of low-volume gymnastics programs conducted under ordinary school conditions remains limited.

Moreover, the majority of previous investigations have been diagnostic or cross-sectional in nature and have primarily relied on clinical or observational assessment methods, without experimentally evaluating the effects of a specifically structured sports gymnastics program on sagittal spinal alignment parameters in younger school-aged children. Furthermore, only a few experimental studies have investigated whether a low-volume gymnastics program adapted to the organizational conditions of primary schools can influence thoracic kyphosis, lumbar lordosis, and physiological sagittal spinal alignment.

This methodological and scientific gap justifies the need for an experimental approach based on precise assessment of sagittal spinal alignment parameters using valid and reliable measurement instruments. Therefore, the present study experimentally examined the effects of a specifically structured additional sports gymnastics program adapted to regular school conditions on thoracic kyphosis, lumbar lordosis, and physiological sagittal spinal alignment using a validated instrumental assessment method.

Previous studies have suggested that gymnastics training may be associated with improvements in neuromuscular control, trunk stabilization, proprioceptive function, and postural coordination. However, these functional mechanisms were not directly assessed in the present study and are presented only as possible explanatory mechanisms reported in the literature. The present investigation focused exclusively on objectively measured changes in thoracic kyphosis, lumbar lordosis, and physiological sagittal spinal alignment.

Accordingly, the aim of this study was to determine the effects of additional sports gymnastics training on thoracic kyphosis, lumbar lordosis, and physiological sagittal spinal alignment in fifth-grade primary school students. It was hypothesized that the additional sports gymnastics program would result in more favorable changes in thoracic kyphosis, lumbar lordosis, and the prevalence of physiological sagittal spinal alignment compared with regular physical education classes alone.

## 2. Materials and Methods

A total of 139 fifth-grade students from the primary schools “Stefan Nemanja” and “Rifat Burdžović Tršo” in Novi Pazar participated in this study. The research employed a quasi-experimental longitudinal design with pre- and post-intervention measurements.

Participants included students who regularly attended physical education classes and had no diagnosed structural spinal disorders or other medical conditions that could affect safe participation in the exercise program. Students who did not attend the final assessment were excluded from the final analysis.

The participants were divided into an experimental group (*n* = 74) and a control group (*n* = 65). Group allocation was performed at the school level (cluster sampling), with students from “Rifat Burdžović Tršo” assigned to the experimental group and students from “Stefan Nemanja” assigned to the control group. This approach was used to maintain organizational consistency and prevent treatment contamination between groups.

The mean age of participants was 11.0 ± 0.5 years, and both sexes were included in the sample. All students participated in regular physical education classes; in addition, the experimental group took part in an extra sports gymnastics training program, while the control group followed handball-based content within the regular curriculum.

The study was approved by the Ethics Committee of the State University of Novi Pazar (approval no. 5471/24). The research was conducted with the approval of school authorities and written informed consent obtained from parents/guardians, in accordance with the ethical principles of the Declaration of Helsinki [29].

### 2.1. Variables and Measurement Instrument

Sagittal spinal alignment and curvature parameters of the spinal column were assessed using the SpinalMouse^®^ (IDIAG M360^®^, Mülistrasse 18, CH-8320 Fehraltorf, Switzerland), a reliable and validated non-invasive instrument for evaluating spinal curvature and spinal mobility without exposure to ionizing radiation [30]. Previous validation studies have demonstrated good-to-excellent reliability and validity of the Spinal Mouse. Post and Leferink [31] reported inter-rater correlation coefficients ranging from r = 0.85 to 0.90 and excellent intraclass correlation coefficients (ICC = 0.92–0.95) for sagittal spinal range-of-motion measurements. Furthermore, Guermazi et al. [32] confirmed the validity and reliability of the instrument for lumbar mobility assessment through comparison with radiographic measurements.

Recent international studies additionally confirmed satisfactory agreement between Spinal Mouse and photogrammetric assessment procedures for sagittal and lumbopelvic alignment evaluation in adolescents with thoracic kyphosis, further supporting its applicability in sagittal spinal assessment and postural analysis [33,34].

It should be emphasized that the Spinal Mouse device primarily evaluates spinal curvature and spinal mobility rather than static posture itself; therefore, the findings obtained in the present study were interpreted in terms of sagittal spinal alignment and sagittal spinal curvature parameters [35].

### 2.2. Measurement Procedure

All measurements were conducted under standardized environmental and testing conditions during regular school hours in the gymnasium facilities of participating schools. Prior to testing, participants received standardized instructions regarding body position and testing procedures in order to minimize measurement variability.

Participants wore minimal sports clothing (shorts) to enable clear identification of anatomical landmarks and unobstructed movement of the measuring device along the spinal column. Each participant assumed a natural upright standing posture, with arms relaxed alongside the body, knees fully extended, and feet positioned approximately hip-width apart.

Before each testing session, the Spinal Mouse device was calibrated according to the manufacturer’s recommendations. Anatomical landmarks were identified and marked using a dermatographic pencil, with the spinous process of the seventh cervical vertebra (C7) used as the starting point and the third sacral vertebra (S3) as the endpoint of measurement.

The examiner positioned the Spinal Mouse device at the C7 level and guided it manually along the midline of the spine toward S3 while maintaining continuous contact with the skin surface and following the natural sagittal curvature of the spinal column. Measurements were performed by trained evaluators experienced in the application of the Spinal Mouse system.

Possible measurement variability associated with anatomical landmark identification, participant positioning, and repeated measurements should be considered as a potential methodological limitation of the assessment procedure.

### 2.3. Data Analysis and Reference Values

The collected data were transferred to dedicated software associated with the Spinal Mouse device, which generated graphical and numerical representations of sagittal spinal alignment and spinal curvature parameters.

The obtained angular values were interpreted according to previously published pediatric spinal alignment recommendations and clinical classification criteria reported in the literature.

Thoracic kyphosis values in the sagittal plane were classified as follows:

Normal: 30–45°;

Grade I hyperkyphosis: 45–55°;

Grade II hyperkyphosis: >56°.

Lumbar lordosis values in the sagittal plane were classified as follows:

Normal: 20–36°;

Grade I hyperlordosis: 37–45°;

Grade II hyperlordosis: >45°.

### 2.4. Statistical Analysis

Differences in the prevalence of sagittal spinal deviations were analyzed using the chi-square (χ^2^) test. Differences between the experimental and control groups were assessed using an independent-samples *t*-test. When assumptions of normality were not fully satisfied, the Mann–Whitney U test was additionally considered. Changes between initial and final measurements within each group were examined using the paired samples *t*-test.

To evaluate the effects of the intervention while controlling for baseline values, analysis of covariance (ANCOVA) was performed with baseline measurements entered as covariates.

Prior to inferential analyses, the normality of distribution of continuous variables was assessed using the Shapiro–Wilk test. Effect sizes were calculated to facilitate interpretation of practical significance and were expressed as Cohen’s d for between-group comparisons, Cohen’s dz for within-group changes, and partial eta squared (η^2^p) for ANCOVA analyses.

Given the quasi-experimental design and the relatively short intervention period, the obtained findings should be interpreted with caution, particularly in light of the generally small effect sizes observed.

The level of statistical significance was set at *p* < 0.05. All statistical analyses were performed using IBM SPSS Statistics software (version 19) [36].

### 2.5. Training Program and Study Organization

Initial measurements were conducted at the beginning of the school year, while final measurements were performed after a six-week intervention period. All measurements were carried out during regular physical education classes using identical procedures.

Measurements were conducted by trained evaluators, including physical education teachers and doctoral-level students.

During the study, all participants attended regular physical education classes twice per week. Additionally, the experimental group participated in an extra sports gymnastics session once per week (60 min) over six weeks. The control group followed standard physical education classes (45 min), including handball-based activities.

This structure was aligned with World Health Organization recommendations [37], which suggest that children should engage in at least 60 min of moderate-to-vigorous physical activity daily.

Additional Sports Gymnastics Program.

Each training session consisted of four parts:

Introductory (basic movements and games);

Preparatory (conditioning and motor development exercises);

Main (apparatus-based exercises);

Final (flexibility exercises).

The program focused on fundamental elements of sports gymnastics, including floor and vault exercises adapted to the participants’ age. The training content was designed to enhance motor abilities, core stabilization, and sagittal spinal alignment. A detailed overview of the program structure and content is provided in Table 1.

## 3. Results

The descriptive characteristics of the participants are presented in Supplementary Table S1. The experimental (*n* = 74) and control (*n* = 65) groups were highly comparable in terms of age, body height, body mass, and body mass index at baseline.

Descriptive statistics and the results of the Shapiro–Wilk normality analysis are presented in Supplementary Table S2. Most variables showed approximately normal distributions, supporting the use of parametric statistical analyses. Lumbar lordosis at baseline in the experimental group showed a slight deviation from normality; therefore, non-parametric procedures were applied where appropriate.

Baseline and follow-up between-group comparisons are presented in Supplementary Table S3. No statistically significant between-group differences were observed for thoracic kyphosis or lumbar lordosis in the unadjusted analyses.

Table 2 presents the results of within-group changes between baseline and follow-up measurements. No statistically significant within-group changes were observed in the experimental group for either thoracic kyphosis (*p* = 0.873) or lumbar lordosis (*p* = 0.065).

In the control group, a statistically significant reduction in thoracic kyphosis was observed (*p* = 0.001), while changes in lumbar lordosis were not statistically significant.

Table 3 presents the results of the analysis of covariance (ANCOVA) performed on follow-up sagittal spinal alignment parameters after controlling for baseline values. A statistically significant baseline-adjusted difference between groups was observed for thoracic kyphosis (F = 5.05, *p* = 0.026). However, the adjusted mean value was lower in the control group (18.74°) than in the experimental group (22.23°). Therefore, this result should be interpreted as a baseline-adjusted between-group difference rather than evidence of a beneficial effect of the gymnastics intervention.

In contrast, no statistically significant differences between the experimental and control groups were observed for lumbar lordosis after adjustment for baseline values.

Table 4 presents the distribution of participants with physiological sagittal spinal alignment and those with sagittal spinal deformities at baseline and follow-up measurements. At baseline, physiological sagittal spinal alignment was identified in 11.5% of participants in the total sample, whereas 88.5% exhibited some form of sagittal spinal deformity. Following the intervention period, the proportion of participants with physiological alignment increased to 41.0%, accompanied by a reduction in the prevalence of deformities. Improvements were observed in both study groups; however, the experimental group demonstrated a greater increase in physiological sagittal spinal alignment (14.9% to 45.9%) compared with the control group (7.7% to 35.4%). An increase in the proportion of participants with physiological sagittal spinal alignment was observed in both groups. Although the increase appeared descriptively greater in the experimental group, these findings should be interpreted cautiously because they do not by themselves demonstrate an intervention effect.

The detailed distribution of sagittal spinal deformity types is presented in Supplementary Table S4. Descriptive changes in the frequency of deformity categories were observed between baseline and follow-up, particularly for the flat thoracic spine. Because no formal statistical analysis of these categorical changes was performed, these findings should be interpreted descriptively only.

Sex-specific descriptive results are presented in Supplementary Table S5. Although descriptive differences were observed between boys and girls, no formal group-by-sex-by-time interaction analysis was performed. Therefore, these findings should be interpreted with caution.

## 4. Discussion

In accordance with the stated aim of the study, the present research examined the effects of an additional sports gymnastics program on sagittal spinal alignment and indicators associated with the prevention of postural disorders in fifth-grade primary school students. It was hypothesized that students participating in the additional gymnastics program would demonstrate more favorable changes in thoracic kyphosis, lumbar lordosis, and the prevalence of physiological sagittal spinal alignment compared with students who attended only regular physical education classes.

The findings showed that certain descriptive changes in sagittal spinal alignment occurred during the intervention period; however, these changes were not consistently statistically significant or clearly attributable to the additional gymnastics program. Direct between-group comparisons did not reveal statistically significant differences for the main continuous outcome variables. After adjustment for baseline values, ANCOVA indicated a statistically significant difference between groups in baseline-adjusted thoracic kyphosis. Nevertheless, because the adjusted mean value was lower in the control group than in the experimental group, this result should not be interpreted as evidence of a superior corrective effect of the gymnastics intervention. Rather, it represents a baseline-adjusted statistical difference whose practical meaning should be considered cautiously within the context of the overall pattern of findings and the relatively short intervention period.

In the experimental group, thoracic kyphosis decreased from 22.50° to 21.85°, while lumbar lordosis decreased from 31.24° to 28.96°. Although both variables demonstrated descriptive reductions, neither change reached statistical significance. Therefore, these findings should be interpreted as non-significant descriptive changes rather than confirmed intervention effects. The reduction in lumbar lordosis did not reach statistical significance (*p* = 0.065) and should therefore be interpreted as a non-significant descriptive change rather than as evidence of an intervention effect.

Interestingly, the control group demonstrated a statistically significant within-group reduction in thoracic kyphosis, from 23.83° to 19.15° (*p* = 0.001). However, this finding should also be interpreted with caution, because within-group analyses do not control for baseline variability and cannot establish a specific effect of the control condition. The observed change may reflect a combination of regular physical education, normal growth and maturation, natural variability in postural assessment, or increased familiarity with the measurement procedure. Similar observations were reported by Bogdanović and Milenković (2008) [38], who emphasized that meaningful postural changes may occur during the early school years as a consequence of accelerated locomotor growth and temporary imbalances between muscular and skeletal development.

The participants in the present study were approximately 11 years of age, which represents a sensitive developmental period characterized by rapid somatic growth, progressive neuromuscular maturation, and ongoing refinement of postural control. During this stage, sagittal spinal alignment may be particularly responsive to both biological and environmental influences. Consequently, some of the observed changes may have resulted from natural developmental processes rather than from the structured exercise program alone. In addition, repeated exposure to the assessment procedures may have increased postural awareness at follow-up, which could have influenced the measured spinal alignment values.

Gymnastics-based exercise is theoretically associated with improvements in trunk stabilization, balance regulation, and movement coordination. However, these mechanisms were not directly evaluated in the present study and, therefore, should be regarded as plausible explanations rather than confirmed mechanisms underlying the observed findings. Horak (2006) [9] emphasized that effective postural control depends on the integration of sensory information, neuromuscular activation, and biomechanical constraints. Similarly, Zemková and Zapletalová (2022) [27] highlighted trunk stability and neuromuscular control as essential components of functional movement, while Zhang and Zheng (2025) [28] reported that trunk stabilization exercises may contribute to improved postural control and movement efficiency.

The gymnastics program implemented in the present study incorporated floor exercises and vault-related activities that required continuous regulation of body position, activation of the trunk musculature, maintenance of balance, and control of the body’s center of mass. Although these exercise characteristics are theoretically consistent with mechanisms known to support postural regulation, their specific contribution to the observed descriptive changes cannot be confirmed because no biomechanical or neuromuscular variables were directly assessed.

The present findings are also consistent with previous investigations reporting beneficial associations between gymnastics participation and postural function. Andreeva et al. (2021) [39] demonstrated that sports characterized by high demands for coordination, balance, and body control were associated with improved postural stability and more efficient regulation of body position. Likewise, Akin (2013) [40] reported favorable effects of gymnastics training on dynamic balance and movement control in children. Nevertheless, direct comparisons should be interpreted cautiously because the intervention duration, participant characteristics, and outcome measures differed substantially from those employed in the present study.

The generally small effect sizes observed across the analyzed variables further suggest that the practical impact of the intervention was modest. This finding is understandable given the relatively short duration of the program and limited weekly training volume. Similar conclusions were reported by Kravchuk et al. (2020) [41], who suggested that longer and more intensive exercise interventions are required to achieve stable structural and functional adaptations in posture during childhood.

Taken together, the present findings suggest that the six-week gymnastics program was insufficient to induce measurable structural adaptations in sagittal spinal alignment. Although descriptive reductions in thoracic kyphosis and lumbar lordosis were observed, these changes did not consistently reach statistical significance. This finding is not unexpected, considering that structural postural adaptations generally require prolonged and repeated training stimuli. Short-term exercise interventions are more likely to influence functional aspects of movement, such as neuromuscular coordination and postural control, than permanent modifications of spinal curvatures.

The present findings are generally consistent with previous studies reporting beneficial associations between structured exercise programs and postural development in children. Biševac et al. (2021) [42] demonstrated that corrective exercise programs may contribute to favorable changes in functional spinal deformities among preschool- and school-aged children, while Paunović et al. (2023) [43] reported that young gymnasts exhibited more favorable postural characteristics and a lower prevalence of spinal deformities than their non-gymnast peers. Although these findings support the theoretical rationale for gymnastics-based exercise, direct comparisons should be interpreted with caution because of differences in study design, participant characteristics, intervention duration, and outcome measures.

The present results should also be considered within the broader context of contemporary lifestyle factors affecting children’s musculoskeletal health. Aleixo et al. (2012) [25] reported that excessive body weight and obesity are associated with an increased prevalence of postural disorders and alterations in sagittal spinal alignment among school-aged children. Together with the growing prevalence of sedentary behavior and insufficient physical activity, these factors reinforce the importance of implementing structured exercise programs within the school environment as part of comprehensive strategies aimed at promoting healthy musculoskeletal development, Malina et al. (2004) [44].

From a practical perspective, the findings suggest that additional gymnastics-based activities can be feasibly incorporated into regular school-based physical activity programs. Although descriptive improvements were observed, particularly in the categorical distribution of physiological spinal alignment, these findings should be interpreted cautiously and cannot be considered definitive evidence that the intervention improved postural development. Consequently, the effectiveness of gymnastics-based exercise for improving sagittal spinal alignment should be confirmed in future studies employing longer intervention periods, larger samples, and comprehensive biomechanical assessments.

The principal scientific contribution of the present study lies in its longitudinal evaluation of a school-based sports gymnastics program using objective quantitative assessment of sagittal spinal alignment in primary school students. Unlike many previous studies that were predominantly cross-sectional or diagnostic, the present investigation provides longitudinal evidence describing changes in sagittal spinal alignment following a structured gymnastics intervention conducted within the regular school setting. Furthermore, the combination of continuous angular measurements and categorical classification offers a more comprehensive evaluation of postural status than reliance on a single methodological approach.

Future studies should investigate the effects of longer gymnastics-based interventions involving larger and more diverse samples while incorporating comprehensive biomechanical, neuromuscular, and functional assessments. Such investigations may provide a more detailed understanding of the mechanisms through which gymnastics-based exercise influences postural development and may help establish evidence-based recommendations for the prevention of postural disorders in school-aged children.

### Study Limitations

Several methodological limitations of the present study should be acknowledged. First, the intervention period lasted only six weeks and consisted of only six additional sports gymnastics sessions. Although descriptive changes in sagittal spinal alignment were observed, this relatively low training dose was likely insufficient to induce stable structural adaptations of the spinal column or more pronounced changes in spinal curvature parameters.

Second, the gymnastics program was relatively limited in scope and focused primarily on floor exercises and vault-related activities. The inclusion of a broader range of gymnastics apparatus, greater training frequency, and a longer intervention period might have produced more pronounced changes in sagittal spinal alignment.

Third, no follow-up assessment was conducted after completion of the intervention; therefore, the long-term sustainability of the observed changes remains unknown. Furthermore, habitual physical activity, extracurricular sports participation, biological maturation, and the content of regular physical education classes were not fully controlled and may have influenced the observed outcomes.

In addition, potential measurement variability associated with the identification of anatomical landmarks and participant positioning during Spinal Mouse assessment should be considered. Although standardized measurement procedures were applied, these methodological factors may have influenced measurement precision.

Finally, the quasi-experimental design and school-based group allocation, without individual randomization, limited the ability to establish causal relationships between the intervention and the observed outcomes. Moreover, functional variables such as trunk stabilization, postural control, proprioception, movement coordination, and neuromuscular regulation were not directly assessed. Therefore, no conclusions regarding these potential mechanisms can be drawn from the present findings, and any explanations referring to such mechanisms should be considered speculative. Future studies should employ randomized controlled designs, longer intervention periods, larger training volumes, and direct assessment of functional outcomes to provide a more comprehensive evaluation of the effects of sports gymnastics on sagittal spinal alignment in school-aged children.

## 5. Conclusions

The findings of the present study suggest that a six-week additional sports gymnastics program was associated with descriptive changes in sagittal spinal alignment among fifth-grade primary school students. Small reductions in thoracic kyphosis and lumbar lordosis were observed in the experimental group; however, these changes did not reach statistical significance.

After adjustment for baseline values, ANCOVA demonstrated a statistically significant between-group difference for thoracic kyphosis. However, because the adjusted mean value was lower in the control group than in the experimental group, this finding should be interpreted cautiously and should not be considered evidence of a superior effect of the gymnastics intervention.

An increase in the proportion of participants classified as having physiological sagittal spinal alignment was observed in both groups, with a descriptively greater increase in the experimental group. These findings suggest that the intervention may have been associated with favorable changes in categorical sagittal spinal alignment; however, the results should be interpreted as preliminary and exploratory because of the quasi-experimental design, the low intervention dose, and the absence of long-term follow-up.

Overall, the present study provides preliminary evidence that a short-term school-based sports gymnastics program may represent a feasible approach for investigating changes in sagittal spinal alignment in school-aged children. Nevertheless, further randomized controlled studies with longer intervention periods, larger training volumes, and direct assessment of functional outcomes are required before firm conclusions regarding the effectiveness of gymnastics interventions can be drawn.

## Figures and Tables

**Table 1:** Structure of the training program in the experimental and control groups.

Week	Session Part	Experimental Group Activities	Control Group Activities
I–II	Introductory	Warm-up through basic locomotor movements or simple games	Basic locomotor movements
	Preparatory	Conditioning exercises to improve motor abilities, including: pull-ups (underhand and overhand grip, 2 × 5 reps), dips on parallel bars (2 × 3), handstand against the wall (10 s facing and back to wall), push-ups (2 × 5)	General conditioning exercises
	Main	Floor and vault training focused on basic movement patterns and safe landing techniques. Floor exercises included forward rolls, push-up landings, and preparatory drills (tucked and extended body positions, rolling in different directions, hand supports, and transitions between positions). Vault exercises emphasized take-off and landing mechanics, body control, and balance through simple two-foot jumps and controlled squat landings	Ball handling, catching, and passing with both hands; one-handed passing
	Final	Cool-down and stretching focusing on arms, shoulders, and hamstrings	Cool-down
III–IV	Introductory	Warm-up through basic movements or simple games	Basic movements or simple games
	Preparatory	Conditioning and motor exercises: leg raises on stall bars (2 × 5), leg lifts on Swedish bench (both legs and alternating, 2 × 5 + 10 s hold), handstand against wall (10 + 10 s)	General conditioning exercises
	Main	Floor training included backward rolls, shoulder stand (“candle”), and backward roll progression with preparatory exercises (backward rolling, pelvic lifting, shoulder support, transitions). Emphasis on body grouping, center of mass control, and coordination. Vault training included straddle vault progression through take-off drills, short approach jumps, and low obstacle simulations. Focus on coordination and controlled landing	Dribbling; defensive stance and movement
	Final	Cool-down and stretching focusing on hamstrings and splits	Cool-down
V–VI	Introductory	Warm-up through basic movements and games	Basic movements and games
	Preparatory	Conditioning exercises: pull-ups (2 × 5), dips (2 × 5), handstand against wall (10 + 10 s), push-ups (2 × 5), leg raises (2 × 5)	General conditioning exercises
	Main	Focus on the integration of learned elements into complete routines on the floor and vault. Floor exercises included side rolls, 180° jump turns, dive rolls, headstand, and balance elements, emphasizing control and fluid transitions. Vault included linking movements (support swings, squat support transitions, forward tucked transitions), emphasizing take-off, flight phase, and controlled landing	Shooting at goal; complex shooting techniques (jump shots in height and distance)
	Final	Cool-down and stretching focusing on upper and lower extremities	Cool-down

**Table 2:** Analysis of within-group changes using the paired samples *t*-test.

Group	Variable	Mean Change	t	*p*	Cohen’s dz	Magnitude
Experimental	Thoracic Kyphosis	−0.21	0.16	0.873	−0.019	Trivial
Experimental	Lumbar Lordosis	−2.07	1.88	0.065	−0.228	Small
Control	Thoracic Kyphosis	−4.97	3.42	0.001	−0.435	Small
Control	Lumbar Lordosis	0.77	−0.56	0.581	0.071	Trivial

Legend: Mean Change—mean difference between follow-up and baseline measurements; t—paired samples *t*-test statistic; Cohen’s dz—effect size for within-group comparisons.

**Table 3:** ANCOVA of follow-up sagittal spinal alignment parameters controlling for baseline values.

Variable	Adjusted Mean (EG)	Adjusted Mean (CG)	F	*p*	Partial η^2^	Magnitude
Thoracic Kyphosis	22.23	18.74	5.05	0.026	0.038	Small
Lumbar Lordosis	28.77	30.72	2.24	0.137	0.017	Small

Legend: Adjusted Mean—mean value adjusted for baseline measurements; F—ANCOVA F statistic; Partial η^2^—effect size expressed as partial eta squared; EG—experimental group; CG—control group.

**Table 4:** Distribution of physiological sagittal spinal alignment and spinal deformities in the total sample and study groups.

Group	Physiological Alignment *n* (%)	Deformity Present *n* (%)	Missing *n* (%)	*p*
Gi (*n* = 139)	16 (11.5)	123 (88.5)	–	<0.001
Gf (*n* = 139)	57 (41.0)	73 (52.5)	9 (6.5)	0.161
Ei (*n* = 74)	11 (14.9)	63 (85.1)	–	<0.001
Ef (*n* = 74)	34 (45.9)	34 (45.9)	6 (8.2)	1.000
Ki (*n* = 65)	5 (7.7)	60 (92.3)	–	<0.001
Kf (*n* = 65)	23 (35.4)	39 (60.0)	3 (4.6)	0.042

Legend: Gi—total sample at baseline; Gf—total sample at follow-up; Ei—experimental group at baseline; Ef—experimental group at follow-up; Ki—control group at baseline; Kf—control group at follow-up.

## Data Availability

The original contributions presented in this study are included in the article. Further inquiries can be directed to the corresponding authors.

## Supplementary Materials

The following supporting information can be downloaded at: https://www.mdpi.com/article/10.3390/jfmk11030270/s1, Table S1: Descriptive Characteristics of Participants by Group; Table S2: Descriptive Statistics and Normality Analysis of Sagittal Spinal Alignment Parameters Using the Shapiro–Wilk Test; Table S3: Between-Group Comparisons of Sagittal Spinal Alignment Parameters Using the Independent Samples *t*-Test and Mann–Whitney U Test; Table S4: Distribution of Sagittal Spinal Deformity Types in the Total Sample and Study Groups; Table S5: Distribution of Physiological Sagittal Spinal Alignment According to Sex.
