# Supplementary material for: Effects of an Additional Sports Gymnastics Program on Sagittal Spinal Alignment and Postural Status in School-Aged Students
Source: J Funct Morphol Kinesiol. 2026 Jul 14;11(3):270. doi: 10.3390/jfmk11030270 (PMC13397986; doi:10.3390/jfmk11030270)
Supplement: Supplementary file 1 [file jfmk-11-00270-s001.zip › jfmk-4389921-supplementary.pdf]

Supplementary Table S1 presents the basic anthropometric characteristics of the participants included in the study, including age, body height, body mass, and body mass index (BMI). The experimental group consisted of 74 students, whereas the control group comprised 65 students.

Table S1. Descriptive Characteristics of Participants by Group.

Group	N	Age (years)	Body Height (cm)	Body Mass (kg)	BMI (kg/m²)
Experimental	74	11.02 ± 0.51	148.3 ± 8.4	46.7 ± 9.2	21.2 ± 3.1
Control	65	11.08 ± 0.47	149.1 ± 7.9	47.4 ± 8.7	21.5 ± 3.3

Legend: N – number of participants; BMI – body mass index. Values are presented as mean ± standard deviation (M ± SD).

Analysis of the presented parameters indicates that participants in both groups were highly comparable with respect to their baseline anthropometric characteristics. The mean age of participants was approximately 11 years in both groups, while mean values of body height, body mass, and BMI were very similar between the experimental and control groups. Such homogeneity of baseline characteristics suggests that the groups were adequately matched prior to the implementation of the experimental program.

Supplementary Table S2 presents the descriptive statistics and results of the normality analysis for thoracic kyphosis and lumbar lordosis values in the experimental and control groups at baseline and follow-up measurements.

Table S2. Descriptive Statistics and Normality Analysis of Sagittal Spinal Alignment Parameters Using the Shapiro–Wilk Test.

Group	Measurement	Variable	Mean ± SD	Min–Max	Shapiro–Wilk	*p*
Experimental	Baseline	Thoracic Kyphosis	22.50 ± 11.19	3–50	0.975	0.141
Experimental	Follow-up	Thoracic Kyphosis	21.85 ± 9.32	2–47	0.991	0.922
Control	Baseline	Thoracic Kyphosis	23.83 ± 9.89	1–48	0.973	0.163
Control	Follow-up	Thoracic Kyphosis	19.15 ± 10.03	0–49	0.975	0.247
Experimental	Baseline	Lumbar Lordosis	31.24 ± 9.33	−4–52	0.967	0.049
Experimental	Follow-up	Lumbar Lordosis	28.96 ± 7.34	12–48	0.969	0.087
Control	Baseline	Lumbar Lordosis	29.17 ± 10.61	3–56	0.988	0.758
Control	Follow-up	Lumbar Lordosis	30.52 ± 8.68	14–50	0.986	0.683

Legend: Mean ± SD – mean ± standard deviation; Min–Max – minimum and maximum values; Shapiro–Wilk – test of normality of distribution; p – statistical significance level.

The results of the Shapiro–Wilk test indicated that most of the analyzed distributions did not significantly deviate from normality ($p > 0.05$), thereby supporting the use of parametric statistical procedures in subsequent analyses. The only exception was lumbar lordosis at baseline in the experimental group, which showed a slight deviation from normal distribution ($p = 0.049$). Therefore, the use of non-parametric procedures was considered appropriate for selected analyses.

In the experimental group, small descriptive reductions in thoracic kyphosis (22.50° to 21.85°) and lumbar lordosis (31.24° to 28.96°) were observed between baseline and follow-up measurements. However, these descriptive changes should be interpreted cautiously because statistical significance was not observed for either variable in subsequent analyses. In the control group, a more pronounced reduction in thoracic kyphosis values was observed (23.83° to 19.15°), whereas lumbar lordosis values showed a slight increase between baseline and follow-up measurements (29.17° to 30.52°).

Supplementary Table S3 presents the results of between-group comparisons for the analyzed sagittal spinal alignment parameters. No statistically significant differences were identified between the experimental and control groups at either baseline or follow-up measurements for any of the analyzed variables (p > 0.05).

Table S3. Between-Group Comparisons of Sagittal Spinal Alignment Parameters Using the Independent Samples t-Test and Mann–Whitney U Test.

Variable	Measurement	Test	Value	p	Effect Size	Magnitude
Thoracic Kyphosis	Baseline	t-test	-0.74	0.462	-0.126	Trivial
Thoracic Kyphosis	Follow-up	t-test	1.60	0.113	0.280	Small
Lumbar Lordosis	Baseline	Mann–Whitney U	2776.50	0.117	0.133	Small
Lumbar Lordosis	Follow-up	t-test	-1.11	0.269	-0.195	Trivial

Legend: t-test – independent samples t-test; Mann–Whitney U – non-parametric test; Effect Size – magnitude of the difference between groups; Magnitude – interpretation of effect size.

The largest between-group difference was observed for thoracic kyphosis at follow-up. However, the difference did not reach statistical significance in the unadjusted analysis.

Supplementary Table S3 presents the results of within-group changes between baseline and follow-up measurements. No statistically significant within-group changes were observed in the experimental group for either thoracic kyphosis (p = 0.873) or lumbar lordosis (p = 0.065).

Supplementary Table S4 shows the distribution of sagittal spinal deformity types at baseline and follow-up. Flat thoracic spine (RLT) was the most common deformity in both groups at baseline. After the intervention, decreases were observed in RLT, TotRL, and LORRLT, particularly in the experimental group. Descriptive changes in the frequency of sagittal spinal alignment categories were observed between baseline and follow-up, particularly for flat thoracic spine. Because no formal statistical comparison of these categorical changes was performed, these findings should be interpreted descriptively only.

Table S4. Distribution of sagittal spinal deformity types in the total sample and study groups.

Group	KIF	RLT	LOR	RLL	TotRL	KIFLOR	LORRLT
Gi	2 (1.4)	60 (43.2)	13 (9.4)	1 (0.7)	20 (14.4)	2 (1.4)	25 (18.0)
Gf	1 (0.7)	38 (27.3)	16 (11.5)	0	9 (6.5)	1 (0.7)	8 (5.8)
Ei	1 (1.4)	31 (41.9)	7 (9.5)	0	8 (10.8)	1 (1.4)	15 (20.3)
Ef	1 (1.4)	20 (27.0)	7 (7.9)	0	3 (4.1)	0	3 (4.1)
Ki	1 (1.5)	29 (44.6)	6 (9.2)	1 (1.5)	12 (18.5)	1 (1.5)	10 (15.4)
Kf	0	18 (27.7)	9 (13.8)	0	6 (9.2)	1 (1.5)	5 (7.7)

Legend: KIF – hyperkyphosis; RLT – flat thoracic spine; LOR – lumbar hyperlordosis; RLL – flat lumbar spine; TotRL – total flat back; KIFLOR – kypholordotic posture; LORRLT – lumbar hyperlordosis with flat thoracic spine.

Supplementary Table S5 presents the distribution of physiological sagittal spinal alignment and sagittal spinal deformities according to sex at baseline and follow-up measurements. Descriptive differences were observed between boys and girls. The largest increase in physiological sagittal spinal alignment was observed among girls in the experimental group. However, no formal group-by-sex-by-time interaction analysis was performed; therefore, these findings should be interpreted with caution. The greatest improvement was recorded among girls in the experimental group, where the prevalence of physiological sagittal spinal alignment increased from 17.6% at baseline to 64.7% at follow-up.

Table S5. Distribution of physiological sagittal spinal alignment according to sex.

Group	Physiological alignment n (%)	Deformity present n (%)	Missing n (%)	p
Mi (n=75)	7 (9.3)	68 (90.7)	–	<0.001
Mf (n=75)	25 (33.3)	44 (58.7)	6 (8.0)	0.022
Ži (n=64)	9 (14.1)	55 (85.9)	–	<0.001
Žf (n=64)	32 (50.0)	29 (45.3)	3 (4.7)	0.701
MiE (n=40)	5 (12.5)	35 (87.5)	–	<0.001
MfE (n=40)	12 (30.0)	24 (60.0)	4 (10.0)	0.046
MiK (n=35)	2 (5.7)	33 (94.3)	–	<0.001
MfK (n=35)	13 (37.1)	20 (57.2)	2 (5.7)	0.223
ŽiE (n=34)	6 (17.6)	28 (82.4)	–	<0.001
ŽfE (n=34)	22 (64.7)	10 (29.4)	2 (5.9)	0.034
ŽiK (n=30)	3 (10.0)	27 (90.0)	–	<0.001
ŽfK (n=30)	10 (33.3)	19 (63.3)	1 (3.3)	0.095

Legend: Mi – male participants at baseline; Mf – male participants at follow-up; Ži – female participants at baseline; Žf – female participants at follow-up; MiE – male participants in the experimental group at baseline; MfE – male participants in the experimental group at follow-up; MiK – male participants in the control group at baseline; MfK – male participants in the control group at follow-up; ŽiE – female participants in the experimental group at baseline; ŽfE – female participants in the experimental group at follow-up; ŽiK – female participants in the control group at baseline; ŽfK – female participants in the control group at follow-up.
